# HTLV-1 Tax disrupts the host epigenome by interacting with a Polycomb group protein EZH2

**DOI:** 10.1186/1742-4690-11-S1-P144

**Published:** 2014-01-07

**Authors:** Dai Fujikawa, Makoto Yamagishi, Naoya Kurokawa, Ai Soejima, Takaomi Ishida, Yuetsu Tanaka, Kazumi Nakano, Toshiki Watanabe

**Affiliations:** 1 Graduate School of Frontier Sciences, The University of Tokyo, Tokyo, Japan; 2 Institute of Medical Science, The University of Tokyo, Tokyo, Japan; 3 Graduate School and Faculty of Medicine, University of the Ryukyus, Okinawa, Japan

## 

ATL is a highly aggressive T-cell neoplasm caused by HTLV-1. We have recently demonstrated that EZH2, a catalytic enzyme of histone H3K27 methylation, is overexpressed in ATL cells, which contributes to persistent NF-κB activation by repressing a tumor-suppressive miRNA, miR-31. This suggests that epigenetic abnormalities are closely associated with the molecular hallmarks of leukemic cells. However, the mechanisms by which epigenetic disruption is caused and sustained in HTLV-1 infected cells remain to be clarified. In the present study, we first found that Tax directly interacts with EZH2 in HTLV-1 infected cells. Given that epigenetic state is directly affected by cell lineage-dependent transcription factors and genetic background, we assessed the functional interconnection between HTLV-1 Tax and the histone methyltransferases in an appropriate model. Using a lentivirus vector, we introduced *tax* gene into peripheral blood mononuclear cells (PBMCs) and CD4+ T-cells from healthy donors and established Tax-transduced T-cells. In this model, we observed overexpression of EZH2, aberrant accumulation of H3K27 trimethylation, and epigenetic silencing of *p21^cip1/waf1^*, all of which have been observed in primary ATL cells. In addition, EZH2 inhibition reduced the viability of Tax-transduced T-cells. Our results strongly suggest that Tax epigenetically affects gene expression through its interaction with EZH2, and that Tax-dependent epigenetic abnormalities may be involved in determining the molecular characteristics of HTLV-1-infected cells as well as ATL cells. Since epigenetic marks are potentially reversible, development of genuine epigenetic-targeted drugs will hold great promise in treatment of HTLV-1-related diseases.

